# Microstructure Evolution of 2A12 Aluminum Alloy under Isothermal Heat Treatment Direct Writing Process

**DOI:** 10.3390/ma15186279

**Published:** 2022-09-09

**Authors:** Wenjuan Rong, Zhongde Shan, Baoyu Wang, Yongwei Wang, Jialin Wang

**Affiliations:** 1 School of Mechanical Engineering, University of Science and Technology Beijing, Beijing 100083, China; 2 State Key Laboratory of Advanced Forming Technology and Equipment, China Academy of Machinery Science and Technology, Beijing 100083, China; 3 College of Mechanical & Electrical Engineering, Nanjing University of Aeronautics and Astronautics, Nanjing 210016, China

**Keywords:** fast manufacturing, metal 3D printing, metal-melting direct-writing forming, semi-solid thermoforming

## Abstract

A metal-melting direct writing process, using semi-solid isothermal heat treatment to form high-quality semi-solid components, realized the integrated innovation of semi-solid formation and additive manufacturing. An experimental study was carried out on semi-solid isothermal heat treatment for metal-melting direct-writing technology, using 2A12 aluminum alloy as raw material. The semi-solid isothermal heat treatment was carried out over different temperature ranges, and four-stages evolution mechanism of the semi-solid microstructure in the semi-solid melting direct writing process was investigated. The effects of holding temperature and time on the microstructure of the semi-solid isothermal heat treatment of the alloy were put forward. According to the analysis of the results of the semi-solid-melting direct-writing test, the corresponding relationship between semi-solid microstructure and extrusion formability was found. The results show that when the holding temperature is 640–650 °C and the holding time is 20–25 min, the liquid phase rate can reach about 40%, and the direct-writing forming technology can be carried out stably.

## 1. Introduction

Semi-solid metal (SSM) forming technology has the characteristics of high forming efficiency, good forming quality, and reduced energy waste, which has been widely valued and studied. The metal parts formed by this technology, especially the aluminum alloy material parts, have broad application prospects due to their high forming accuracy and good mechanical properties [1]. Scholars worldwide have made active efforts in theoretical research as well as its use and promotion. However, the use of semi-solid aluminum alloys is limited to casting, forging, and rolling [2,3,4,5].

Metal-melting direct-writing technology is a rapid metal manufacturing technology. It was first put forward in China by the R & D team of the China Academy of Machinery Science and Technology. This method deposits liquid metal layer by layer and channel by channel on the substrate, according to the preset path to obtain 3D metal parts, which have the characteristics of high efficiency, low cost, flexibility, and green properties. At present, it has been widely used in the rapid and direct manufacturing of high-performance and complex structural parts in the fields of automotive and construction machinery [6,7]. Based on direct 3D-printing forming technology for metal parts, the R & D team applied the melting direct writing process to semi-solid aluminum alloy forming, using semi-solid isothermal heat treatment to produce high-quality semi-solid billet, and continuous extrusion for direct-writing formation, to realize the integrated innovation of semi-solid forming and additive manufacturing [8]. This process combines the advantages of low cost and high efficiency of direct-writing formation with the characteristics of easy-machining, high-performance, and reduced-characteristics of semi-solid metal, which can lead to higher accuracy and better quality for the product parts.

The formation performance and quality of the semi-solid direct writing process mainly depend on the internal microstructure changes in of semi-solid metal [9,10]. In contrast, the microstructure evolution process of the semi-solid billet is directly related to the temperature and length of semi-solid isothermal treatment. Therefore, it is crucial to the control of the high-quality direct-writing forming process control to accurately grasp the change law of the semi-solid organization in the isothermal treatment process and establish the corresponding relationship between the isothermal treatment–microstructure–direct writing forming process. For the evolution law of the semi-solid microstructure, the methods proposed by predecessors at present are relatively simple for reproducing and summarization of the whole dynamic evolution process. Li [11] has proposed effects of short-time isothermal treatment on the microstructure characteristics, element distribution, grain stability, and coarsening kinetics of semi-solid CuSn10P1 alloy. However, the evolution process, except for coarsening, has not been studied. Mohammad [12] studied semi-solid microstructures of 7075 aluminum alloy during the reheating process. However, the change in evolution mechanism over time has not been discussed. Therefore, it is of great significance to study the change law of the whole dynamic evolution process.

In this paper, the 2A12 aluminum alloy is taken as the semi-solid direct-writing research object due to its wide application and good material behavior. Different semi-solid temperature ranges we selected to obtain the evolution law of semi-solid microstructure; the direct-writing formability of the microstructure under different conditions was analyzed, and a suitable heating temperature and holding time we selected. Through the extrusion direct writing process after isothermal heat treatment, semi-solid direct-writing formation was carried out, and the influence law and mechanism of process parameters on forming quality were studied.

## 2. Materials and Methods

The test used a semi-solid isothermal treatment method to prepare a semi-solid billet for a direct-writing formation. The semi-solid isothermal heat treatment method is to first deform the cast deformation aluminum alloy billet above the recrystallization temperature thermal deformation, then to cool this to room temperature and apply direct heat to the semi-solid state temperature insulation [13]. There were many dislocation entanglements and plugs in the deformed aluminum alloy billet after hot working, as well as a high number of subgrain boundaries, which then undergo secondary dynamic recrystallization while heating from room temperature to semi-solid temperature [14,15]. Furthermore, the microstructure changes from elongated crystal to equiaxed crystal to spherulite, from which high-quality semi-solid billets can be prepared. After the semi-solid temperature isothermal treatment, the extruded parts were directly written into shapes, which significantly shortened the process. Coupled with the low deformation resistance of semi-solid billet and its good shape-maintenance ability without external force, this provided technical advantages in the formation high-precision direct-writing forming parts.

**Figure 1:**
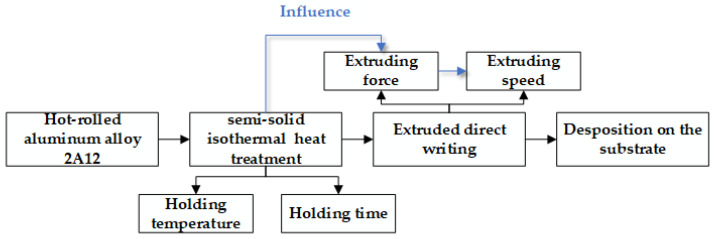
Scheme of the semi-solid direct-writing formation process.

In this experiment, the pre-denaturation and pretreatment steps were omitted, and the hot-rolled aluminum alloy 2A12 sheet was used to further simplify the semi-solid direct writing process. The scheme of the semi-solid direct-writing formation process is shown in Figure 1.

The hot-rolled aluminum alloy sheets can be cut according to the volume and margin of the parts formed by direct writing, which have the characteristics of a short process and low cost, and are suitable for industrial production. The typical chemical components of the 2A12 aluminum alloy used for the experiments are shown in Table 1.

According to the solidus and liquidus temperatures of the 2A12 alloy, the corresponding semi-solid temperature range was obtained, and then the material was heated to reach this holding temperature range [16]. According to the results in the literature, the chemical components of material, holding temperature and holding time are the most important factors affecting the evolution of the semi-solid microstructure in the semi-solid isothermal heat treatment [17,18]. In this paper, the chemical components are not discussed and will be studied in the follow-up works. The water quenching was carried out after the set process conditions were reached, the microstructure of the specimen was retained during isothermal treatment for observation and analysis, and the influence of the temperature and holding time of the isothermal treatment on the evolution of the semi-solid structure of the hot-rolled 2A12 aluminum alloy was studied.

**Figure 2:**
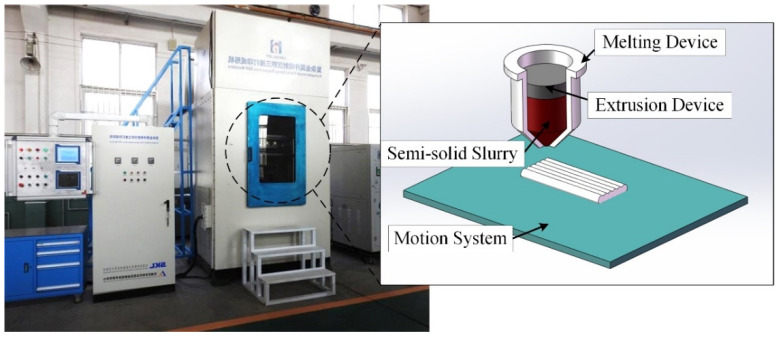
Semi-solid direct-writing forming equipment diagram.

The direct-writing forming process test was carried out on the self-developed semi-solid aluminum alloy melting direct-writing equipment (SMDW-3000, China Academy of Machinery Science and Technology, Beijing, China) as shown in Figure 2, which includes three parts: a melting device, extrusion device, and motion system. The solidus temperature and liquidus temperature of the used alloy were 564.29/693.31 °C, which were determined by using a differential scanning calorimeter (DSC3, Mettler-Toledo, Greifensee, Switzerland). Preliminary tests with different holding temperatures and holding times were carried out. The microstructure images with obvious differences, obtained in the preliminary test, were selected to determine the test parameters. The semi-solid isothermal heat treatment parameters are selected as shown in Table 2, and the direct-writing forming process test was carried out to obtain the required aluminum alloy parts specimen. The microstructure images were acquired using the Digital Microscope (VXH-700, KEYENCE, Osaka, Japan) with optical shadow effect mode and Emission Scanning Electron Microscope (Gemini SEM-500, Zeiss, Oberkochen, Germany).

## 3. Results

### 3.1. Semi-Solid Isothermal Heat Treatment Process and Microstructure Analysis

#### 3.1.1. Effects of Holding Temperature on the Semi-Solid Microstructure

**Figure 3:**
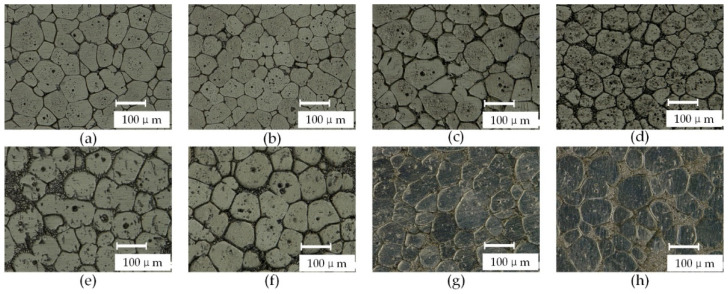
Semi-solid microstructure under isothermal heat treatment for 20 min, (**a**) 610 °C, (**b**) 620 °C, (**c**) 630 °C, (**d**) 640 °C, (**e**) 650 °C, (**f**) 660 °C, (**g**) 670 °C, and (**h**) 680 °C.

Figure 3 shows the microtissue of the isothermal treatment test ranging from 610 °C to 680 °C for 20 min. When the isothermal treatment temperature is 610~620 °C, the crystal particles in the microstructure have polygonal granular appearance. The spherical process is not apparent; the liquid phase appears at the triangular crystal boundary, gradually infiltrates into the crystal boundary, and gradually increases the liquid phase. As the temperature increases, the spherical trend is more pronounced. The effect of grain spheroidization is ideal when the temperature is above 640 °C. The effect of grain spheroidization is ideal when the heat preservation temperature is between 640 and 680 °C. Varying with the temperature, the degree of spheroidization has little difference.

**Figure 4:**
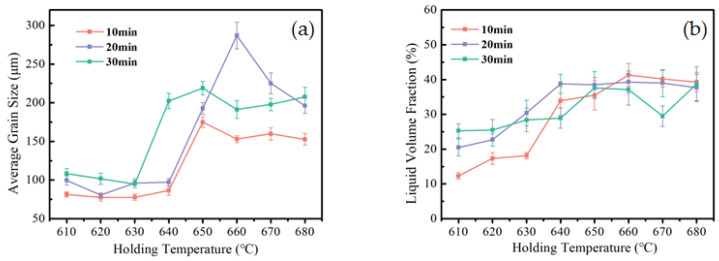
Quantitative analysis of microstructure at different holding temperatures; (**a**) average grain size, and (**b**) liquid volume fraction.

Figure 4a,b show the liquid volume fraction and average grain size, calculated using the metallographic analysis software ImageJ. When the holding temperature increases from 610 °C to 640 °C, the liquid volume fraction in the semi-solid billet is increased from 10~25% to 30~40%, and 640 °C is the critical holding temperature of the 2A12 aluminum alloy semi-solid microstructure, which changes from a low to a high liquid volume fraction. The holding temperature continues to improve, and the liquid volume fraction gradually stabilizes at about 40%. The grain size did not change much when the holding temperature ranged from 610 to 630 °C. The average size of the primary α-Al phase slightly decreased due to the infiltration of the liquid melting phase. When the holding temperature increased from 630 to 650 °C, the average size of the primary α-Al phase slightly decreased due to the infiltration of the liquid melting phase. When the heat preservation temperature increased from 630 to 650 °C, the average grain size of the primary α-Al phase significantly increased, and when the heat preservation temperature reached 650 to 680 °C, the average grain size of the primary α-Al phase did not obviously change with the increase in temperature.

#### 3.1.2. Effects of Holding Time on the Semi-Solid Microstructure

**Figure 5:**
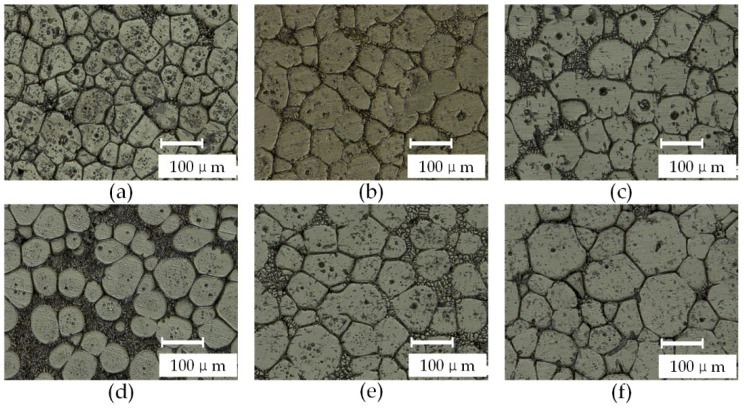
Microstructure of isothermal heat treatment at a different time when holding temperature is 650 °C, (**a**) 10 min, (**b**) 15 min, (**c**) 20 min, (**d**) 25 min, (**e**) 30 min, and (**f**) 35 min.

Figure 5 shows the semi-solid isothermal treatment microstructure with a holding time of from 10 min to 35 min under the holding temperature of 650 °C. When the sample holds its temperature for 10~15 min, most of the low melting point phases at the grain boundary of the primary α-Al grains begin to melt, and the grain boundary becomes relatively clear. Some adjacent grains condense and grow, resulting in a significant increase in the grain size. Irregular granular crystals can still be observed, and the phenomenon of grain sphere crystallization is not significant. When the holding time increased to 20~25 min, the remelt phase significantly increased, and was mainly distributed around the grains; this resulted in a significant decrease in the number of condensation growth grains and an increase in the roundness of grains. The holding time increased to 30~35 min, while the solid phase particles grew and coarsened.

**Figure 6:**
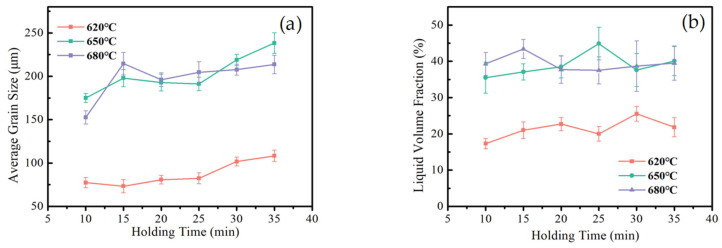
Quantitative analysis of microstructure at different holding times; (**a**) average grain size, and (**b**) liquid volume fraction.

In the semi-solid holding temperature range, the change of liquid phase volume shows a relatively gentle trend with the change in temperature [19,20]. Comparing Figure 4a with Figure 6a, the liquid volume fraction is less sensitive to the change of holding time than to the change in holding temperature. This provides a basis for the regulation of semi-solid-melt direct-writing process control methods. To ensure the rapid and accurate control of the liquid volume fraction, the holding temperature should be paid more attention to in the semi-solid-melt direct writing process.

According to Figure 6, as the holding time increases, the liquid phase melting volume slowly increases; over time, the grains coarsened, occupying the liquid phase volume, and the liquid volume fraction showed a trend of first increasing and then decreasing. As the holding temperature increases, the critical time needed for the liquid volume fraction to change from increasing to decreasing becomes higher. When the holding temperature is 620 °C, the coarsening critical time is 30 min; when the holding temperature is raised to above 650 °C, the coarsening critical temperature is advanced to 20 min.

### 3.2. Mechanism of Semi-Solid Microstructure Evolution

With the increase in holding temperature or the extension of holding time, α-Al grain presents significantly different shapes. Each characteristic microstructure represents a different semi-solid grain evolution mechanism.

**Figure 7:**
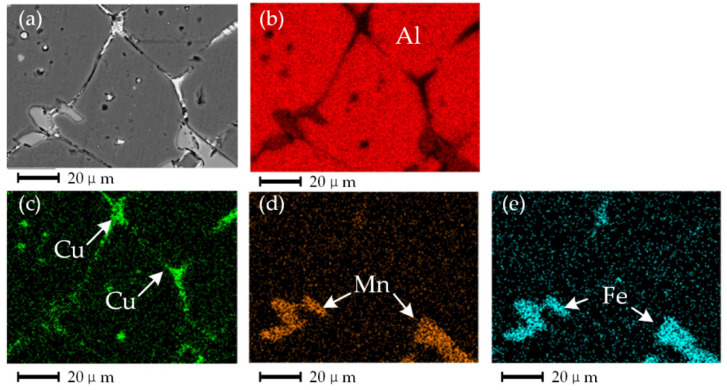
Results of the energy dispersive spectrum analysis, (**a**) microstructure when holding temperature is 620 °C and holding time is 10 min, (**b**) distribution of Al, (**c**) distribution of Cu, (**d**) distribution of Mn, and (**e**) distribution of Fe.

Stage I: the melting stage of the eutectic structure. At the beginning of isothermal heat treatment or when the temperature is low, the second phase of the eutectic structure with a low melting point at the grain boundary first begins to melt to form a liquid phase [21]. As shown in Figure 7c, the contrast in the liquid phase is bright because the second phase element, Cu, accumulates in the liquid phase at the crystal boundary [22]. Elements such as Fe and Mn have diffused and migrated to the grain boundary during the heat preservation process and dissolved into the liquid phase, as shown in Figure 7c,d. At this stage, the liquid phase is first formed in the triangular region where the crystal boundary intersects and continues to melt along with the crystal boundary penetration, and the molten liquid phase forms a mesh connection structure along the crystal boundary, surrounding the α-Al grain. At this time, the proportion of the liquid phase is meager, and the α-Al grain presents an irregular granular shape.

**Figure 8:**
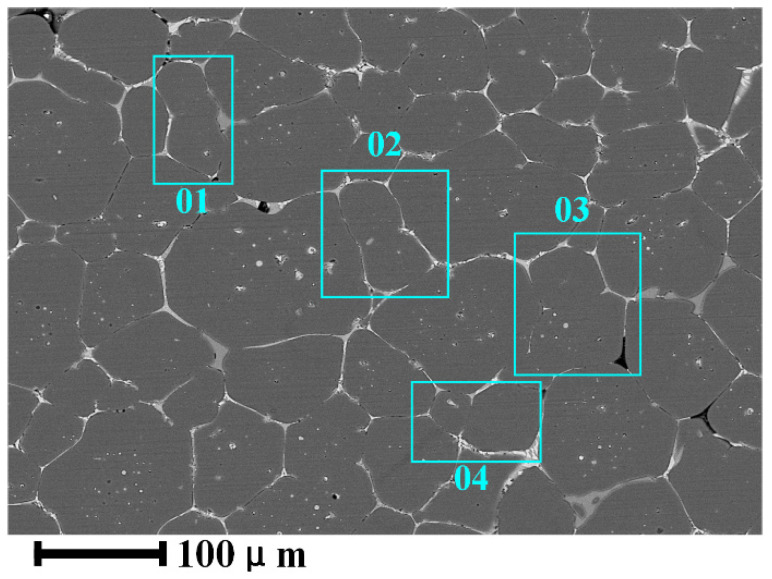
“8”-shaped grains.

Stage II: the stage of grains’ condensation, growth and collision. The system’s free energy increases with the holding time or temperature. Due to thermal convection, the grains begin to move, rotate, and collide in the liquid phase. As there is a certain liquid phase at this stage, the liquid volume fraction is small, and the grains are more likely to collide. The α-Al grains with a similar lattice orientation easily condense under the liquid-solid interface’s tension, reducing the system’s free energy. The grains are primarily in the “8” shape at this stage, as shown in Figure 8. The collision, fusion, and growth of α-Al grains can be observed, and the grain grows more quickly [23,24]. At the same time, due to the large number of defects and distortions in the thermal deformed aluminum alloy crystal boundary and the subcrustal boundary, the crystal boundary diffusion activation energy is low, therefore, after the grain condensation, the liquid phase continues to penetrate along the crystal boundary. The liquid volume fraction continues to grow.

**Figure 9:**
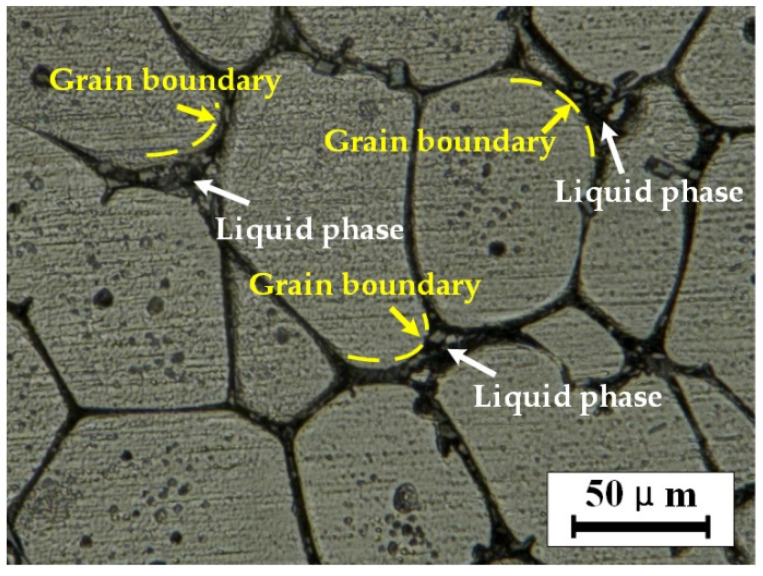
The high radius of curvature component is preferentially melting.

Stage III: spheroidization stage of α-Al grains. With the increase in holding temperature or extension of holding time, the liquid volume fraction increases to more than 40%. The liquid phase is immersed into the primary α-Al grains of the solid phase, increasing the relative distance between grains, and it is more difficult for the coagulation between grains to grow. At this stage, the grain growth mechanism becomes an Ostwald growth. According to the diffusion limited aggregation (DLA) model, the melting point of the large curvature on the grain is low, so melting is preferred [25]. Therefore, the corners of the α-Al grains are first melted into the liquid phase, as shown in Figure 9. At the same time, due to the effect of liquid-solid phase interface tension, according to the principle of minimum surface area, the primary α-Al grains evenly grow around, the solute atoms migrate to the low radius of curvature and deposit them, and the α-Al grains gradually become smoother. At this stage, grain Ostwald grew up slowly, but the degree of particle roundness increased and changed to near-spherical and spherical shapes [26]. The higher the holding temperature, the longer the holding time, and the higher the degree of grain spheronization.

**Figure 10:**
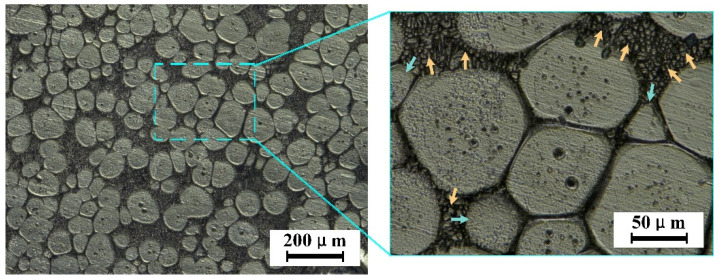
Coarsening of the α-Al grains.

Stage IV: solid-phase particle growth and coarsening stage. The liquid volume fraction gradually reaches the theoretical equilibrium value with the increase in holding temperature or the extent of holding time. The region between the primary α-Al grains is filled with the liquid phase, the spherical grains become independent, and the collision and condensation phenomenon disappears. At this time, the grain growth is realized entirely by the diffusion deposition of atoms in the Ostwald mechanism [27]. After Stage III, α-Al grain roundness is high, but there is a size difference. At this time, Ostwald diffusion and deposition are reflected by the melting or even disappearance of tiny α-Al grains, and large grains continue to grow; that is the coarsening process of the grains, as shown in Figure 10. At this stage, the liquid volume reaches the essential equilibrium, and due to grain growth, part of the liquid volume is squeezed, and the liquid volume fraction is slightly reduced.

**Figure 11:**
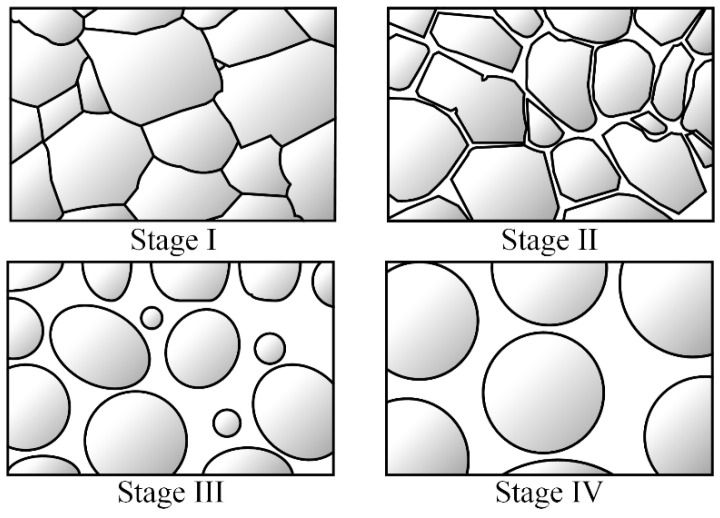
Schematic diagram of the semi-solid-state evolution process.

In summary, the transition process of semi-solid alloy can be divided into four stages, Stage I: the melting stage of the eutectic structure; Stage II: the stage of grains condensation growth and collision; Stage III: spheroidization stage of α-Al grains; Stage IV: solid-phase particle growth and coarsening stage. The main evolution mechanism of the semi-solid microstructure from the first stage to the fourth stage is shown in Figure 11.

## 4. Discussion

**Figure 12:**
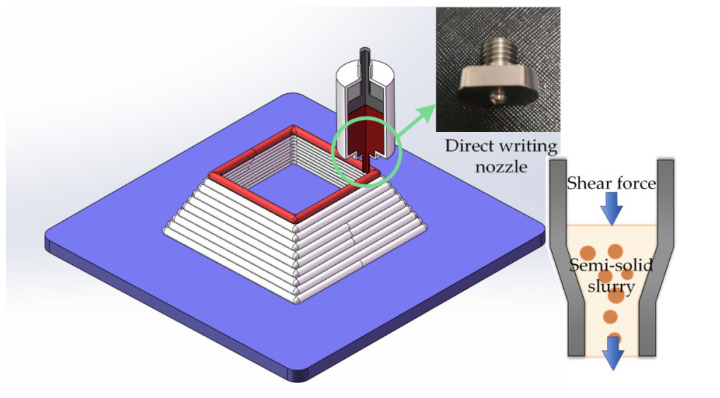
The semi-solid direct writing process.

Semi-solid metals have unique forming advantages in the direct-writing formation process. The semi-solid slurry with a certain solid fraction can support its weight when deposited on the substrate, and the sample will not be deformed and collapsed in the process of layer-by-layer accumulation. The equipment can provide an extrusion force of 0~10 kN, and the semi-solid metals in the crucible are extruded by the piston to generate a uniform shear force that allows for the materials to pass through the crucible-nozzle channel as shown in Figure 12. When subjected to shear force, the deformation resistance of semi-solid metal rapidly decreases, and it has a high fluidity, almost equal to that of liquid metal [28]. Therefore, extrusion allows for the semi-solid metal to smoothly flow out of the direct writing nozzle.

**Figure 13:**
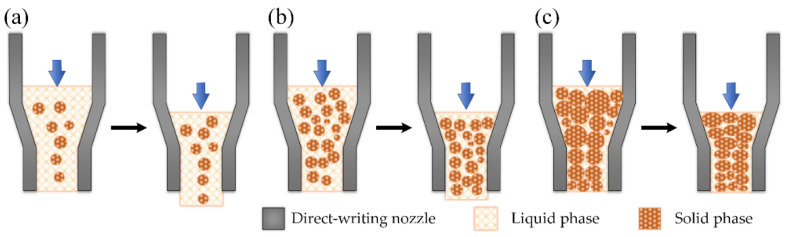
Schematic diagram of melting direct-writing principle, (**a**) high liquid volume fraction, (**b**) liquid-solid mixed flow, and (**c**) low liquid volume fraction.

**Figure 14:**
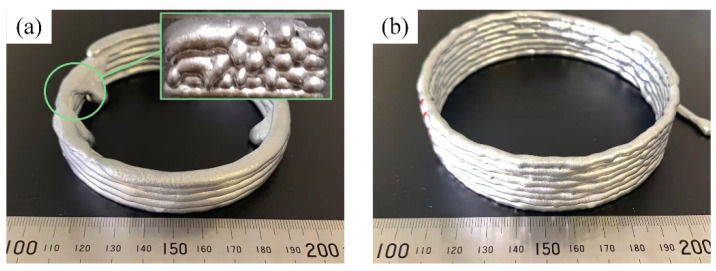
Semi-solid melt direct-writing specimens with different liquid phase volume fractions, (**a**) liquid melting direct writing; (**b**) 35~45% liquid volume fraction semi-solid melt direct writing.

As the liquid fraction changes from high to low, the semi-solid deformation has three main forms:

1. Liquid-phase flow. The liquid phase surrounds the solid phase particles, each independent of the other as shown in Figure 13a. After compression, the solid phase particles gather in the center, and the liquid phase flows to the edge and gradually solidifies after the deformation. At this time, the deformation of the semi-solid metal is close to liquid, and the deformation extrusion force is tiny. However, the formation trajectory will deform and collapse when deposited on the substrate because the solid phase composition is too little to support its weight. The forming specimen is shown in Figure 14a.
2. Liquid-solid phase mixed flow. During deformation, the solid phase particles are surrounded by the liquid phase and flow independently of each other with the liquid phase as shown in Figure 13b. At this time, the pressure can overcome the constraints and friction caused by the space around the solid phase particles, and the required extrusion pressure is slight [29]. At the same time, the solid phase particles play a skeleton role after extrusion, supporting the direct-writing metal to maintain the track shape, which is not easy to collapse when it accumulates between the layers. The formation specimen is shown in Figure 14b.
3. Slide movement and deformation between solid-phase particles. At this high solid-phase fraction condition, the solid phase particles in the semi-solid metal are relatively close, and the flow is mainly realized by the crystal boundary slip between the solid phase particles, as shown in Figure 13c.

The deformation process should overcome the friction between solid-phase particles and the yield stress of solid-phase-particle deformation, so the highest extrusion force is required.

According to the characteristics of the semi-solid forming process, semi-solid isothermal treatment test research was carried out, to obtain the corresponding relationship between the temperature and the liquid volume fraction of the semi-solid slurry. The forming parameters window (the holding temperature and time) for selection was formulated. On the basis of the experiments, the liquid volume fraction should be kept at about 35~45% to maintain a continuous and uniform extrusion direct-writing formation.

For semi-solid melt direct writing, there are three criteria for selecting a semi-solid liquid volume fraction: a semi-solid slurry with a higher solid fraction is beneficial to maintain the size characteristics of direct writing, and the first derivative of the solid fraction to temperature is small so that the sensitivity of the liquid fraction to temperature is small enough to ensure that semi-solid direct writing has process stability. A specific liquid volume fraction and stable liquid-solid mixing flow can be achieved during the extrusion of direct writing. Furthermore, there should be a smaller grain size to improve the mechanical properties of the samples [30,31]. Formation parameters with a holding temperature of 640–650 °C and a holding time of 20–25 min were selected to obtain a liquid volume fraction that reaches about 40%, achieving a uniform and stable direct writing formation.

## 5. Conclusions

An experimental study on semi-solid isothermal heat treatment was carried out for the metal melting direct writing process. The effects that holding temperature and time have on the microstructure of semi-solid isothermal heat treatment of direct writing were put forward. The following conclusions can be obtained:

(1) A melting direct-writing semi-solid metal process based on isothermal heat treatment is proposed, and the correspondence between the isothermal treatment parameters and the semi-solid microstructure of 2A12 aluminum alloy is established.
(2) The evolution mechanism of the semi-solid microstructure under isothermal treatment conditions is proposed to realize the reconstruction of a microstructure change law in the semi-solid melting direct writing process.
(3) After the semi-solid melting direct-writing test study, a corresponding relationship between the semi-solid microstructure and the extrusion-forming performance is obtained. The liquid-solid phase mixed-flow mechanism provides favorable conditions for the semi-solid melting direct-writing forming.
(4) The semi-solid isothermal treatment process parameters that are suitable for 2A12 aluminum-alloy direct writing were selected. When the holding temperature is 640–650 °C and the holding time is 20–25 min, the liquid volume fraction reaches about 40%, achieving a uniform and stable direct-writing formation.

## Figures and Tables

**Table 1:** Typical chemical components of 2A12 aluminum alloy.

Element	Cu	Mg	Mn	Fe	Si	Zn
Wt/%	0.83~1.9	0.21~1.8	≤0.3	≤0.5	≤0.5	≤0.3

**Table 2:** Process test parameters.

Tests	Holding Temperature (°C)	Holding Time (min)
Temperature tests	610, 620, 630, 640, 650, 660, 670, 680	20
Time tests	650	10, 15, 20, 25, 30
